# Epidemiology, risk factors and outcomes of *Candida albicans vs.* non-*albicans* candidaemia in adult patients in Northeast China

**DOI:** 10.1017/S0950268819001638

**Published:** 2019-09-25

**Authors:** Wei Zhang, Xingpeng Song, Hao Wu, Rui Zheng

**Affiliations:** Department of Respiratory Medicine, Shengjing Hospital of China Medical University, No. 36 Sanhao Street, Heping District, Shenyang, Liaoning, 110004, China

**Keywords:** *Candida albicans*, candidaemia, mortality, non-*albicans Candida*, risk factor

## Abstract

This study aimed to evaluate the clinical characteristics, risk factors and outcomes of adult patients with candidaemia caused by *C. albicans vs.* non-*albicans Candida* spp. (NAC). All adult hospitalised cases of candidaemia (2012–2017) at a tertiary hospital in Shenyang were included in the retrospective study, and a total of 180 episodes were analysed. *C. parapsilosis* was the most frequently isolated species (38.3%), followed by *C. albicans* (35.6%), *C. glabrata* (13.9%), *C. tropicalis* (10%) and others (2.2%). As initial antifungal therapy, 75.0%, 3.9%, 5.6% and 2.2% of patients received fluconazole, caspofungin, micafungin and voriconazole, respectively. Multivariate analyses revealed that total parenteral nutrition was associated with an increased risk of NAC bloodstream infections (BSI) (OR 2.535, 95% CI (1.066–6.026)) *vs. C. albicans* BSI. Additionally, the presence of a urinary catheter was associated with an increased risk of *C. albicans* BSI (OR 2.295 (1.129–4.666)) *vs.* NAC BSI. Moreover, ICU stay (OR 4.013 (1.476–10.906)), renal failure (OR 3.24 (1.084–9.683)), thrombocytopaenia (OR 7.171 (2.152–23.892)) and *C. albicans* (OR 3.629 (1.352–9.743)) were independent risk factors for candidaemia-related 30-day mortality, while recent cancer surgery was associated with reduced mortality risk (OR 26.479 (2.550–274.918)). All these factors may provide useful information to select initial empirical antifungal agents.

## Introduction

*Candida* is an important causative organism of bloodstream infections (BSIs). Over the last two decades, candidaemia has been reported as the fourth and seventh most common healthcare-associated BSI in US and European studies, respectively [1, 2]. In a recent multicentre point-prevalence survey, *Candida* species emerged as the most common bloodstream pathogen and accounted for up to 22% of healthcare-associated BSIs [3]. Additionally, candidaemia remains associated with high-mortality rates, prolonged hospital stays and increased healthcare costs [1, 4–7]. Overall, mortality rates among patients range from 19.6% to 67% worldwide [1, 6, 8–12] and the major risk factors for candidaemia include receipt of parenteral nutrition, exposure to broad spectrum antibiotics, presence of central venous catheter (CVC), prior surgery and ICU stay [13, 14]. Even though *C. albicans* overall accounts for the majority of *Candida* spp. causing candidaemia, the proportion of non-*albicans Candida* (NAC) spp. is rising and has even exceeded that of *C. albicans* in some recent studies from Europe, Asia and America [8–10, 15–17].

In general, the most frequently isolated NAC spp. from candidaemia cases include *C. glabrata*, *C. parapsilosis*, *C. tropicalis and C. krusei* [5, 10, 16, 17]. Reduced in vitro susceptibility to antifungal agents has been observed among several NAC spp., which may present a therapeutic challenge. *C. glabrata* and *C. krusei* tend to be less susceptible to azole agents than other *Candida* spp., while *C. parapsilosis* shows the highest minimum inhibitory concentrations (MICs) to the echinocandins than other *Candida* spp. [18, 19]. Several retrospective studies suggest that delayed initiation of antifungal therapy after the onset of candidaemia is associated with increased mortality, which highlights the importance of early appropriate antifungal therapy [20]. The local epidemiology and variable antifungal susceptibility profiles of different *Candida* spp. are critical for the selection of antifungal therapy prior to culture and susceptibility data being available.

However, risk factors related to NAC BSI, and the distribution and antifungal susceptibility of *Candida* spp. from BSI differ geographically, whereas data on risk factors, antifungal susceptibility and outcomes in *C. albicans* and NAC BSIs remain scarce in Northeast China. Therefore, this retrospective study was performed to compare clinical characteristics and outcomes between *C. albicans* and NAC candidaemia, analyse prognostic factors and determine risk factors related to *C. albicans* or NAC at a tertiary hospital in Shenyang.

## Patients and methods

### Subjects and study design

This retrospective observational study was undertaken from January 2012 to October 2017 at a tertiary grade A comprehensive hospital in Shenyang, China. The setting is a China Medical University-affiliated teaching hospital with over 6000 beds currently. All adult hospitalised cases of candidaemia were included in the study. The following data were collected from medical records: age, sex, major underlying diseases and predisposing factors, surgical and invasive procedures, complications, blood tests, isolated *Candida* spp., antifungal susceptibility, antifungal therapy, duration of hospitalisation and final outcome. The study was approved by the Medical Ethics Committee in Shengjing Hospital of China Medical University (reference number 2018PS506K).

### Microbiological methods

Blood samples were drawn under sterile conditions and processed using a BD BACTEC 9120 or BD BACTEC FX400 (Becton Dickinson Diagnostic Instrument Systems, Sparks, USA) blood culture system. After the identification of *Candida* isolates utilising the VITEK 2 YST (bioMérieux, Durham, USA) card, antifungal susceptibility testing of fluconazole, itraconazole, voriconazole and amphotericin B was determined using a ATB FUNGUS 3 (BIOMERIEUX) strip following the manufacturer's instructions. The interpretation of antifungal susceptibility of fluconazole, itraconazole, voriconazole and amphotericin B against *C. albicans*, *C. glabrata*, *C. parapsilosis*, *C. tropicalis* and *C. krusei* was performed by applying clinical breakpoints (CBPs) defined by the Clinical Laboratory Standards Institute (CLSI) [21] or European Committee on Antimicrobial Susceptibility Testing (EUCAST) [22]. MIC CBPs for echinocandins and 5-fluorocytosine by the CLSI or EUCAST were not available at the time, and therefore are not reported in the current study.

### Clinical definitions

An episode of candidaemia was defined as the isolation of a *Candida* spp. recovered from the first blood culture in a patient with accompanying clinical signs and symptoms. Subsequent positive cultures from the same patient were considered as a new episode if the interval between the two episodes was more than 30 days. The onset of candidaemia was taken as the day the first positive blood culture for *Candida* spp. was drawn from the patient. Except for recent surgery, defined as y within 3 months, the predisposing factors occurred within 30 days prior to the onset of candidaemia. Elderly patients were defined as those 65 years of age or older. Laboratory test results were obtained at the onset of candidaemia. Renal failure was defined as a serum creatinine above 104 µmol/l. Anaemia was defined as a haemoglobin level below 110 g/l for women and 130 g/l for men. Hypoalbuminaemia was defined as albumin <30 g/l and hyponatraemia as a serum sodium concentration <135 mmol/l. Leukopaenia was defined as a peripheral white blood cell count less than 4 × 10^9^/l and thrombocytopaenia as a platelet count <100 × 10^9^/l. Antifungal therapy was defined as empirical if started before susceptibility test results were obtained and as targeted thereafter. The outcome was registered after 30 days from the onset of candidaemia.

### Statistical analysis

Continuous variables were expressed as the mean ± standard deviation in normally distributed data or as the median and interquartile range (25th–75th percentiles) in data with non-normal distributions, respectively. Categorical variables were reported as number (%). Continuous variables were compared using the Student's *t*-test or Mann–Whitney *U*-test according to the normality of distribution. Categorical variables were compared using the *χ*^2^ test or *χ*^2^ continuity correction test. Variables with *P*-values of ⩽0.10 including age, sex, underlying diseases and predisposing factors were entered into the multivariable logistic regression model to identify factors associated with NAC *vs. C. albicans* candidaemia. All variables with *P*-values of ⩽0.10 in the univariate analysis of factors associated with mortality were included in the multivariate logistic regression model to identify independent factors associated with mortality. Two-sided *P*-values of <0.05 were considered statistically significant. Statistical analyses were carried out with SPSS (IBM SPSS Statistics 21.0).

## Results

During the study period, 188 episodes of candidaemia in 187 adult patients were initially identified. Two patients were excluded from further analysis because they had a single positive blood culture for *Candida* spp. without signs and symptoms of infection. One blood culture was considered to be contaminated due to simultaneous isolation of *Staphylococcus epidermidis* with *C. glabrata*. Five patients were lost to follow-up within 30 days from the onset of candidaemia and were therefore excluded. Thus, the remaining 180 episodes of candidaemia from 179 adult patients were included in the study. Over the study period, 74 patients developed candidaemia in the first 3-year period (2012–2014) (74/2845, 26.0/1000), which did not evidently differ from cases per hospitalisation-days in the second 3-year period (2015–2017) (106/3769, 28.1/1000) (*P* = 0.611).

### Distribution of *Candida* spp. causing bloodstream infection

**Fig. 1.**
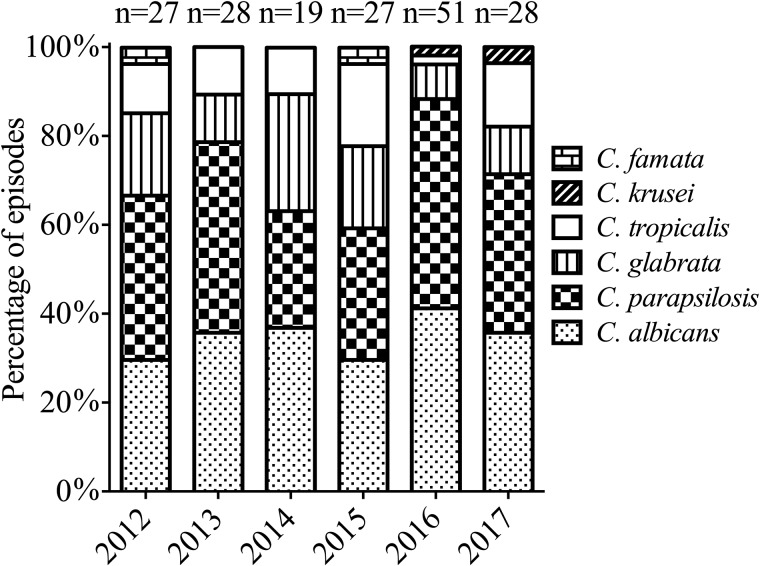
Distribution of *Candida* species isolates during the study period.

Among the 180 *Candida* isolates from blood cultures, *C. parapsilosis* was the predominant species (69/180, 38.3%), followed by *C. albicans* (64/180, 35.6%), *C. glabrata* (25/180, 13.9%), *C. tropicalis* (18/180, 10%), *C. krusei* (2/180, 1.1%) and *C. famata* (2/180, 1.1%). Mixed infections with two or more *Candida* spp. were not observed. Figure 1 shows the distribution of the *Candida* spp. from candidaemia cases from 2012 to 2017. NAC accounted for the majority and there were no substantial changes regarding species distribution patterns between *C. albicans* and NAC over the study period. The proportion of *C. parapsilosis* BSI in surgical patients evidently increased compared with non-surgical patients (59/138 (42.8%) *vs.* 10/42 (23.8%), *P* = 0.027) (Table 1).

**Table 1.** Distribution of *Candida* species among patients according to prior surgery or history of solid tumours

Species	Non-surgical patients (42)	Surgical patients (138)	*P*	Non-solid tumours (100)	Solid tumours (80)	*P*
*C. albicans*	19 (45.2%)	45 (32.6%)	0.134	36 (36%)	28 (36%)	0.889
*C. parapsilosis*	10 (23.8%)	59 (42.8%)	0.027	35 (35%)	34 (35%)	0.304
*C. glabrata*	8 (19.0%)	17 (12.3%)	0.270	15 (15%)	10 (12.5%)	0.630
*C. tropicalis*	4 (9.5%)	14 (10.1%)	1.0	13 (13%)	5 (6.3%)	0.134

### Patient characteristics

The mean age of all episodes was 61.8 ± 15.9 years and 122/180 (67.8%) were male. *C. albicans* and NAC spp. were responsible for 64/180 (35.6%) and 116/180 (64.4%) cases, respectively. Among surgical patients, 138/180 (76.7%) episodes were documented. Other common predisposing factors for candidaemia included prior antibiotics exposure (163/180, 90.6%) and total parenteral nutrition (TPN) (152/180, 84.4%). Nonetheless, there were no statistical differences between *C. albicans* and NAC cases for the proportion of surgical patients and those who received prior antibiotics treatment. At the onset of candidaemia, 148/180 (82.2%) patients presented with anaemia. Moreover, the overall low frequency of fluconazole resistance to *Candida* BSI isolates (12/180, 6.7%) is noteworthy.

The comparison of demographics and clinical data of patients with *C. albicans vs.* NAC candidaemia is summarised in Table 2. NAC-infected patients were more likely to have hyponatraemia than those infected with *C. albicans* (50/116 (43.1%) *vs.* 18/64 (28.1%), *P* = 0.047). Likewise, significant differences between NAC and *C. albicans* patients with BSI were evident for the presence of a urinary catheter (68/116; 58.6% and 47/64; 73.4% *P* = 0.048), the in vitro susceptibility of isolates to fluconazole (72/116; 62.1% and 57/64; 89.1% *P* < 0.001) and the 30-day mortality (23/116; 19.8% and 23/64; 35.9% *P* = 0.018). Other statistically significant elevated differences between the two groups were the frequency of current and former smokers (40/116; 34.5%, and 14/64; 21.9% *P* = 0.077) and TPN (102/116; 88%, and 50/64; 78.1%, and *P* = 0.082).

**Table 2.** Demographics and clinical characteristics of patients with *C. albicans* and non-*albicans* candidaemia

Characteristics	*C. albicans n* = 64 (35.6%)	Non-*albicans n* = 116 (64.4%)	*P* Value
Age	61.3 ± 15.1	62.2 ± 16.5	0.726
Male sex	41 (64.1%)	81 (69.8%)	0.428
Underlying diseases			
Haematological malignancy	3 (4.7%)	4 (3.4%)	0.993
Solid tumours	28 (43.8%)	52 (44.8%)	0.889
Diabetes mellitus	16 (25%)	25 (21.6%)	0.597
Predisposing factors			
Dialysis	2 (3.1%)	6 (5.2%)	0.795
Current and former smokers	14 (21.9%)	40 (34.5%)	0.077
ICU stay	19 (29.7%)	37 (31.9%)	0.759
Surgical patients	45 (70.3%)	93 (80.2%)	0.134
Recent abdominal surgery	35 (54.7%)	73 (62.9%)	0.280
Recent cancer surgery	25 (39.1%)	46 (39.7%)	0.938
Prior antibiotics exposure	60 (93.8%)	103 (88.8%)	0.276
Prior antifungal exposure	8 (12.5%)	18 (15.5%)	0.581
TPN	50 (78.1%)	102 (87.9%)	0.082
Presence of CVC	28 (43.8%)	40 (34.5%)	0.220
Mechanical ventilation	3 (4.7%)	9 (7.8%)	0.632
Presence of urethral catheter	47 (73.4%)	68 (58.6%)	0.048
Presence of gastric tube	42 (65.6%)	80 (69%)	0.646
Laboratory findings			
Renal failure	13 (20.3%)	25 (21.6%)	0.845
Anaemia	49 (76.6%)	99 (85.3%)	0.140
Hypoalbuminaemia	26 (40.6%)	44 (37.9%)	0.723
Hyponatraemia	18 (28.1%)	50 (43.1%)	0.047
Leukopaenia	9 (14.1%)	16 (13.8%)	0.960
Thrombocytopaenia	10 (15.6%)	18 (15.5%)	0.985
Fluconazole susceptibility	57 (89.1%)	72 (62.1%)	<0.001
Outcome			
Crude 30-day mortality	23 (35.9%)	23 (19.8%)	0.018
LOS (days)	28 (21–38.3)	30 (20–48)	0.469

ICU, intensive care unit; TPN, total parenteral nutrition; CVC, central venous catheter; LOS, length of hospital stay.

Multivariate analysis (Table 3) confirmed that TPN was significantly associated with an increased risk of NAC candidaemia (odds ratio (OR) 2.535; 95% confidence interval (CI) 1.066–6.026; *P* = 0.035), whereas the presence of a urinary catheter was distinctly associated with an increased risk of *C. albicans* infection (OR 2.295; CI 1.129–4.666; *P* = 0.022). Current and former smokers were more associated with NAC candidaemia (OR 1.824; CI 0.885–3.756; *P* = 0.103), but the trend did not reach statistical significance.

**Table 3.** Multivariate logistic regression analysis of factors associated with an increased risk of candidaemia due to NAC species *vs. C. albicans*

Risk factors	*B*	s.e.	Wald	*P* Value	OR (95% CI)
Current and former smokers	0.601	0.369	2.655	0.103	1.824 (0.885–3.756)
TPN	0.93	0.442	4.432	0.035	2.535 (1.066–6.026)
Presence of urinary catheter	−0.831	0.362	5.263	0.022	0.436 (0.214–0.886)
Constant	0.205	0.426	0.232	0.63	1.227 (–)

*B*, coefficient; s.e., standard error; Wald test statistic; OR, odds ratio; CI, confidence interval; TPN, total parenteral nutrition.

### Antifungal susceptibility testing

Table 4 shows that of the 180 *Candida* isolates tested for antifungal susceptibility, 12 (6.7%) exhibited resistance to fluconazole, including *C. albicans* (2/64; 3.1%), *C. parapsilosis* (2/69; 2.9%), *C. tropicalis* (6/18; 33.3%) and both isolates of *C. krusei*. Overall, voriconazole resistance was uncommon (10/180; 5.6%) and except for one isolate of *C. glabrata*, all other *Candida* isolates were susceptible to amphotericin B. According to the species-specific CBPs, all 25 isolates of C. *glabrata* showed susceptible-dose dependence (SDD) to fluconazole.

**Table 4.** In vitro antifungal susceptibility of *Candida* species isolated from 180 candidaemia episodes

Species		MIC (μg/ml)			No. (%) of isolates by new CBPs^a^		
(*n* = 180)	Antifungal agent	Ranges	MIC 50	MIC 90	*S*	SDD/*I*	*R*
*C. albicans*	Fluconazole	⩽0.5–16	⩽1	1	57 (89.1%)	5 (7.8%)	2 (3.1%)
(*n* = 64)	Voriconazole	⩽0.03–4	⩽0.06	0.125	57 (89.1%)	3 (4.7%)	4 (6.3%)
	Itraconazole	0.062–1	⩽0.125	0.125	NA	NA	NA
	Amphotericin B	⩽0.25–1	⩽0.5	0.5	64 (100%)	0	0
*C. parapsilosis*	Fluconazole	⩽0.5–8	⩽1	⩽4	61 (88.4%)	6 (8.7%)	2 (2.9%)
(*n* = 69)	Voriconazole	⩽0.03–1	⩽0.06	0.125	66 (95.7%)	2 (2.9%)	1 (1.4%)
	Itraconazole	⩽0.062–0.25	⩽0.125	0.125	67 (97.1%)	0	2 (2.9%)
	Amphotericin B	⩽0.25–1	⩽0.5	0.5	69 (100%)	0	0
*C. glabrata*	Fluconazole	⩽1–16	4	16	0	25 (100%)	0
(*n* = 25)	Voriconazole	0.06–2	0.125	0.25	NA	NA	NA
	Itraconazole	⩽0.125–1	0.25	1	NA	NA	NA
	Amphotericin B	⩽0.25–2	⩽0.5	0.5	24 (96%)	0	1 (4%)
*C. tropicalis*	Fluconazole	⩽1–⩾128	2	16	11 (61.1%)	1 (5.6%)	6 (33.3%)
(*n* = 18)	Voriconazole	⩽0.06–8	0.125	4	12 (66.7%)	2 (11.1%)	4 (22.2%)
	Itraconazole	⩽0.125–8	0.125	2	9 (50%)	0	9 (50%)
	Amphotericin B	⩽0.25–0.5	⩽0.5	0.5	18 (100%)	0	0
*C. krusei*^b^^,^^c^	Fluconazole	64–128			0	0	2 (100%)
(*n* = 2)	Voriconazole	0.5–>8			1 (50%)	0	1 (50%)
	Itraconazole	1–>4			NA	NA	NA
	Amphotericin B	1			2 (100%)	0	0
*C. famata*^b^	Fluconazole	⩽1–4			NA	NA	NA
(*n* = 2)	Voriconazole	⩽0.06–0.125			NA	NA	NA
	Itraconazole	⩽0.125–0.125			NA	NA	NA
	Amphotericin B	⩽0.25–⩽0.5			NA	NA	NA

a Except lack of C. *famata* specific CBPs, CBPs for *Candida* susceptibility to fluconazole and voriconazole were obtained from CLSI [21], while CBPs for susceptibility of *Candida* against itraconazole and amphotericin B were from EUCAST [22].

b MIC 50 and 90 values were not calculated for antifungal drugs against *C. krusei* and *C. famata* due to the small number of *C. krusei* and *C. famata* cases.

c Isolates of *C. krusei* are considered to be intrinsically resistant to fluconazole. S, susceptible; I, intermediate; SDD, susceptible-dose dependent; *R*, resistant; NA, non-applicable.

### Therapy, outcomes and risk factors associated with mortality

The majority of patients (135/180, 75.0%) received fluconazole as first line treatment; caspofungin, micafungin and voriconazole were prescribed initially in 7/180 (3.9%), 10/180 (5.6%) and 4/180 (2.2%) of episodes, respectively. The overall 30-day mortality rate was 25.6% and for patients receiving either targeted, empirical, or no antifungal therapy, the 30-day mortality rates were 24.3% (9/37), 24.6% (29/118) and 32.0% (8/25), respectively, with no statistical difference between the therapy groups (*P* = 0.728). However, the proportion of patients who had not received antifungal therapy did not significantly differ for those who died or survived within 30 days.

On univariate analysis (Table 5), a lower proportion of surgical patients (*P* = 0.085), and an increased proportion receiving mechanical ventilation (*P* = 0.096) were observed in patients dying within 30 days, although not statistically significant. By multivariate regression analysis, four independent risk factors for death were identified: ICU stay (OR 4.013; CI 1.476–10.906; *P* = 0.006), renal failure (OR 3.24; CI 1.084–9.683; *P* = 0.035), thrombocytopaenia (OR 7.171; CI 2.152–23.892; *P* = 0.001) and the isolation of *C. albicans* (OR 3.629; CI 1.352–9.743; *P* = 0.011), while recent cancer surgery was associated with decreased mortality (OR 0.038; CI 0.004–0.392; *P* = 0.006) (Table 6).

**Table 5.** Univariate analysis of factors associated with 30-day mortality of patients with candidaemia

Characteristics	Survivors *n* = 134 (74.4%)	Deaths *n* = 46 (25.6%)	*P* Value
Age	61.5 ± 15.3	63 ± 17.8	0.570
Male sex	90 (67.2%)	32 (69.6%)	0.764
Underlying diseases			
Haematological malignancy	3 (2.2%)	4 (8.7%)	0.130
Solid tumours	67 (50%)	13 (28.3%)	0.010
Diabetes mellitus	30 (22.4%)	11 (23.9%)	0.831
Predisposing factors			
Dialysis	7 (5.2%)	1 (2.2%)	0.652
Current and former smokers	42 (31.3%)	12 (26.1%)	0.502
ICU stay	25 (18.7%)	31 (67.4%)	<0.001
Surgical patients	107 (79.9%)	31 (67.4%)	0.085
Recent abdominal surgery	88 (65.7%)	20 (43.5%)	0.008
Recent cancer surgery	63 (47%)	8 (17.4%)	<0.001
Prior antibiotics exposure	119 (88.8%)	44 (95.7%)	0.281
Prior antifungal exposure	20 (14.9%)	6 (13%)	0.754
TPN	112 (83.6%)	40 (87%)	0.586
Presence of CVC	42 (31.3%)	26 (56.5%)	0.002
Mechanical ventilation	6 (4.5%)	6 (13%)	0.096
Presence of urethral catheter	88 (65.7%)	27 (58.7%)	0.395
Presence of gastric tube	95 (70.9%)	27 (58.7%)	0.127
Laboratory findings			
Renal failure	19 (14.2%)	19 (41.3%)	<0.001
Anaemia	109 (81.3%)	39 (84.8%)	0.599
Hypoalbuminaemia	42 (31.3%)	28 (60.9%)	<0.001
Hyponatraemia	60 (44.8%)	8 (17.4%)	0.001
Leukopaenia	16 (11.9%)	9 (19.6%)	0.197
Thrombocytopaenia	10 (7.5%)	18 (39.1%)	<0.001
*C. albicans*	41 (30.6%)	23 (50%)	0.018
Fluconazole susceptibility	99 (73.9%)	30 (65.2%)	0.261
Empirical antifungal therapy	89 (66.4%)	29 (63%)	0.678
No antifungal therapy	17 (12.7%)	8 (17.4%)	0.426
Outcome			
LOS (days)	30 (21–48)	27.5 (16–41.3)	0.191

ICU, intensive care unit; TPN, total parenteral nutrition; CVC, central venous catheter; LOS, length of hospital stay.

**Table 6.** Multivariate logistic regression analysis of risk factors associated with 30-day mortality of patients with candidaemia

Risk factors	*B*	s.e.	Wald	*P* Value	OR (95% CI)
Solid tumours	1.845	0.975	3.581	0.058	6.329 (0.936–42.778)
ICU stay	1.389	0.51	7.417	0.006	4.013 (1.476–10.906)
Surgical patients	0.793	0.658	1.451	0.228	2.209 (0.608–8.02)
Recent abdominal surgery	0.069	0.647	0.011	0.915	1.072 (0.302–3.805)
Recent cancer surgery	−3.276	1.194	7.53	0.006	0.038 (0.004–0.392)
Presence of CVC	0.476	0.491	0.939	0.333	1.61 (0.615–4.216)
Mechanical ventilation	0.521	0.98	0.283	0.595	1.683 (0.247–11.48)
Renal failure	1.176	0.559	4.429	0.035	3.24 (1.084–9.683)
Hypoalbuminaemia	0.861	0.474	3.303	0.069	2.366 (0.935–5.991)
Hyponatraemia	−0.988	0.538	3.376	0.066	0.372 (0.13–1.068)
Thrombocytopaenia	1.97	0.614	10.292	0.001	7.171 (2.152–23.892)
*C. albicans*	1.289	0.504	6.543	0.011	3.629 (1.352–9.743)
Constant	−3.491	0.729	22.947	<0.001	0.03 (–)

*B*, coefficient; s.e., standard error; Wald test statistic; OR, odds ratio; CI, confidence interval; ICU, intensive care unit; CVC, central venous catheter.

## Discussion

The distribution of *Candida* spp. associated with candidaemia varies greatly in different regions of the world. In recent years, some studies [8–10, 15–17] have observed a shift from the more antifungal susceptible type species *C*. *albicans* towards NAC spp., and this is corroborated by the findings of this 6-year study where NAC isolates accounted for the majority (64.4%) of all adult candidaemia episodes. Likewise, we found *C. parapsilosis* to be the most prevalent NAC species (38.3%) which accords with a report from Mexico of 37.9% in 398 patients of all ages during a 3-year surveillance program from five hospitals [23]. Moreover, there are several other reports of *C. parapsilosis* as the most frequent NAC species (20–25%) from blood cultures in Spain [5, 19], Italy [15], Latin America [24] and Shanghai, China [9, 25]. The second most common NAC spp. in the current study was *C. glabrata* (13.9%), which has also been commonly recorded in studies from the USA [10], the UK [13], Greece [26], Beijing, China [17] and Taiwan [27]. In contrast, *C. tropicalis* ranked as the third most common in our series (10%) but was the most frequent NAC isolate in other studies in China, notably Nanjing [28], Chongqing [8], Shanghai [29], Shandong [30] and Taiwan [31]. The reasons for the varied species distribution and frequency remain unclear but patient demographics and underlying medical conditions are most likely contributory factors [16, 19]. The higher frequency of *C. parapsilosis* BSI recorded here in surgical patients clearly warrants further investigation.

Several possible risk factors have been previously identified for the development of NAC candidaemia and include prior antifungal exposure, malignancy, immunosuppressive therapy, abdominal surgery [32], presence of CVCs [26], artificial surgical implants [17], head trauma and bacterial sepsis [29]. Our finding of an association of an indwelling urinary catheter and a higher risk of BSI caused by *C. albicans vs.* NAC is consistent with other studies [33]. Multivariate analysis also confirmed that TPN was associated with an increased risk of NAC spp., compared with *C. albicans* spp., but this is in contrast with Chow *et al*. [34] who linked TPN with a decreased risk of BSI caused by NAC spp., *vs. C. albicans*. In the current study, *C. parapsilosis* accounted for 59.5%, of all NAC isolates whereas in Chow's series [34], *C. glabrata* (49%) was more than twice as frequent as *C. tropicalis* (19%) or *C. parapsilosis* (18%), which may explain the different findings. Frequent usage of TPN has been linked with *C. parapsilosis* candidaemia compared with *C. albicans*, as well as a negative association with *C. tropicalis* [35]. Therefore, we speculate that the possible reason for divergent views may be the variable proportion of *Candida* spp. in the local epidemiological setting.

Smoking increases susceptibility to a wide range of bacterial and viral infections [36] as well as oral candidiasis in HIV infected adults [37]. Based on pooled data from contemporary cohort studies in the USA, the risk of death from infections in current smokers is more than twice that of individuals who have never smoked [38]. However, data on invasive candidiasis and candidaemia in adult smokers remain scarce. Here, we explored the role of smoking in NAC and *C. albicans* BSI. Although, by multivariate analysis, current and former smoking was not independently associated with the occurrence of NAC BSI, it was likely to be a predictive factor for NAC candidaemia as according to our data current and former smokers had an increased risk for NAC BSI, but no significant relationship was found between smoking and death due to candidaemia. More epidemiological, clinical and mechanistic approaches are needed to study further the impact of smoking in such patients.

*C. albicans* blood isolates showed significantly greater susceptibility to fluconazole (89.1%) than NAC isolates (62.1%) (*P* < 0.001) by applying the species-specific new CBPs. In contrast to the overall high-fluconazole susceptibility rate of *C. parapsilosis* (88.4%) and *C. tropicalis* (61.1%) recorded here, some studies from other cities in China have reported relatively reduced fluconazole susceptibility in their corresponding *Candida* spp. For example, a survey from Nanjing reported that only 74.2% of *C. albicans*, 57.7% of *C. parapsilosis*, 9.1% of *C. glabrata* and 31.6% of *C. tropicalis* were susceptible to fluconazole [28]. Based on the CBPs, all 25 isolates of *C*. *glabrata* in this study exhibited SDD to fluconazole, which supports the recommendations from the updated guideline for the management of candidiasis that transition to higher-dose fluconazole should be considered for patients with fluconazole-susceptible C. *glabrata* isolates [14]. We identified only two patients with *C. krusei*, one of which was resistant to voriconazole; each was effectively treated with micafungin and caspofungin, respectively.

Treatment of candidaemia is increasingly problematic owing to accumulated resistance of *Candida* isolates to antifungal agents, especially fluconazole worldwide [24, 39]. Considering safety and efficacy aspects of treatment, echinocandins are now recommended especially in the early treatment of candidaemia, by both USA and European guidelines [14, 39]. In the current study, fluconazole was the most frequently used antifungal agent for primary therapy, followed by echinocandins and voriconazole, although amphotericin B exhibited excellent in vitro activity overall against *Candida* spp. The plausible explanations for the prevalent use of fluconazole are the species distribution pattern of isolates, their high rates of susceptibility of *C. albicans* and *C. parapsilosis* to the agents, and its safety and tolerability compared with amphotericin B, as well as a significantly lower cost. Due to the lack of echinocandin susceptibility testing in the present study, these agents were used as initial empirical therapy for critically ill patients or those considered likely to have a fluconazole-resistant *Candida* spp. based on the recommendations of the USA and European guidelines. [14, 39]

Several studies have reported discrepant results between outcomes of candidaemia and *C. albicans* spp. in comparison with NAC spp. Differences in mortality were not statistically significant in patient cohorts in the USA (58% *vs.* 57%) [34], Beijing (34.7% *vs.* 38.6%) [17] and Shanghai (37.3% *vs.* 27.9%) [29]. Here, we found that *C. albicans* BSI was an independent risk factor associated with the mortality rate compared with NAC BSI (OR 3.629; CI 1.352–9.743; *P* = 0.011), which mirrors data from Shandong and Nanjing in China [8, 30]. By contrast, in Greece, Dimopoulos *et al*., [26] reported NAC candidaemia to be associated with higher mortality than *C. albicans* in non-immunosuppressed, non-neutropenic patients after ICU admission (OR 6.7; 95% CI 1.2–37.7; *P* = 0.03), which could be attributed to inappropriate or delayed therapy owing to the slower growth of NAC isolates on primary culture; patients with NAC candidaemia may also have been more critically ill than those with C. *albicans*.

Mortality rates of candidaemia have been attributed to the relative virulence of different *Candida* spp., a failure of host-defence mechanisms, patient's underlying diseases and complications, inappropriate or delayed treatment and other factors [5, 20, 40]. In animal models, *C. parapsilosis* and *C. krusei* exhibit less virulence than *C. albicans*, *C. tropicalis* and *C. glabrata* [40]. A meta review of dozens of studies concluded in terms of both overall and attributable mortality that *C. tropicalis* and *C. glabrata* are associated with the highest mortality (40 ± 70%), followed by *C. krusei* and *C. albicans* (20 ± 40%), the lowest being *C. parapsilosis* [40]. Accordingly, the predominance of *C. parapsilosis* in our study may have partly contributed to the observed lower mortality in patients with NAC.

Apart from *C. albicans*, the other variables strongly associated with mortality were ICU stay, renal failure and thrombocytopaenia, which suggest that the more severely ill patients had a worse prognosis. Additionally, almost all clearance of candidaemia in survivors who did not receive antifungal treatment occurred in surgical patients following the removal of an indwelling CVC or drainage catheter. Our results indicated that recent cancer surgery was associated with a higher probability of survival but an explanation for this remains unclear. Cancer patients who were judged reasonably able to tolerate surgery, often lacked distant metastases and had less disease severity and fewer complications. Such host factors may therefore be contributory to a reduced mortality rate for recent cancer surgery, and may aid clinicians better to judge the prognosis of these patients with candidaemia.

Our study has several main limitations. Firstly, owing to its retrospective cohort design, factors such as the management of CVC, the use of appropriate antifungal treatments, immunosuppressive, cancer and glucocorticoid therapy, and underlying chronic lung and heart disease, were not evaluated owing to incomplete medical data acquisition. Secondly, in vitro echinocandin susceptibility testing of *Candida* isolates was not performed, and thirdly, the relatively small sample size may impact on the confidence intervals and analysis of risk factors. Lastly, the study was limited to a single centre and thus, the results may not be applicable to other settings.

In summary, over the last 6 years, NAC was predominant among *Candida* isolates from adult candidaemia at Shengjing Hospital in Shenyang. TPN was associated with an increased risk of developing NAC candidaemia compared with *C. albicans* and patients with a urinary catheter were clearly at an increased risk of BSI due to *C. albicans*. Four independent risk factors for candidaemia-related death were identified, namely, ICU stay, thrombocytopaenia, the isolation of *C. albicans* as a significant predictor of survival and recent cancer surgery.
